# RNA Interference by Cyanobacterial Feeding Demonstrates the *SCSG1* Gene Is Essential for Ciliogenesis during Oral Apparatus Regeneration in *Stentor*

**DOI:** 10.3390/microorganisms9010176

**Published:** 2021-01-15

**Authors:** Wei Wei, Chuanqi Jiang, Xiaocui Chai, Juyuan Zhang, Cheng-Cai Zhang, Wei Miao, Jie Xiong

**Affiliations:** 1 Key Laboratory of Aquatic Biodiversity and Conservation, Institute of Hydrobiology, Chinese Academy of Sciences, Wuhan 430072, China; weiwei10081009@163.com (W.W.); jiangchuanqi@ihb.ac.cn (C.J.); chaixiaocui@ihb.ac.cn (X.C.); zhangjuyuan@ihb.ac.cn (J.Z.); cczhang@ihb.ac.cn (C.-C.Z.); miaowei@ihb.ac.cn (W.M.); 2 Shenzhen Institute of Guangdong Ocean University, Shenzhen 518120, China; 3 Guangdong Provincial Engineering Research Center for Aquatic Animal Health Assessment, Shenzhen 518120, China; 4 Shenzhen Public Service Platform for Evaluation of Marine Economic Animal Seedings, Shenzhen 518120, China; 5 CAS Center for Excellence in Animal Evolution and Genetics, Kunming 650223, China; 6 State Key Laboratory of Freshwater Ecology and Biotechnology of China, Wuhan 430072, China

**Keywords:** ciliate, novel RNAi method, *Synechocystis* sp. PCC6803, oral apparatus regeneration

## Abstract

In the giant ciliate *Stentor coeruleus*, oral apparatus (OA) regeneration is an experimentally tractable regeneration paradigm that occurs via a series of morphological steps. OA regeneration is thought to be driven by a complex regulatory system that orchestrates the temporal expression of conserved and specific genes. We previously identified a *S. coeruleus*-specific gene (named *SCSG1*) that was significantly upregulated during the ciliogenesis stages of OA regeneration, with an expression peak at the stage of the first OA cilia appearance. We established a novel RNA interference (RNAi) method through cyanobacteria *Synechocystis* sp. PCC6803 feeding in *S. coeruleus*. The expression of *SCSG1* gene was significantly knocked down by using this method and induced abnormal ciliogenesis of OA regeneration in *S. coeruleus*, suggesting that *SCSG1* is essential for OA regeneration in *S. coeruleus*. This novel RNAi method by cyanobacterial feeding has potential utility for studying other ciliates.

## 1. Introduction

The giant ciliated protozoan, *Stentor coeruleus*, is a tractable model for studying regeneration at the single-cell level. Regeneration of the oral apparatus (OA) and holdfast, and normal shape reconstitution have all been described in *S. coeruleus* [1]. Thereinto, OA regeneration is an attractive and tractable regeneration paradigm in *S. coeruleus*. The OA is the predominant structure on the anterior end of the cell. It consists of thousands of long cilia organized into membranelles that compose the membranellar band (MB) [2]. In *S. coeruleus*, OA shedding can be induced by short urea treatment [3]; after removal and washing, the cells can completely regenerate the OA and finally restore the feeding ability within 9 h, progressing through a series of well-characterized morphological steps [1,4,5]. Single-cell transcriptome analysis revealed that the morphological steps in OA regeneration are driven by a complex regulatory system that includes centriole assembly, ciliogenesis, signaling, cell cycle regulation, transcription, and RNA binding in *S. coeruleus* [4]. Using proteomic identification, we identified 882 OA-associated proteins in extracts from the shedding OA of *S. coeruleus*; most were highly and continuously expressed in OA regeneration [5]. However, few studies so far have focused on the function of genes involved in OA regeneration.

In many non-model ciliates, RNAi is used to study gene function, and RNAi methodologies have been established for *Paramecium* [6], *Euplotes* [7], *Oxytricha* [8], and *Stylonychia* [9] species. Such RNAi studies have commonly used *Escherichia coli* HT115, an RNase Ш-deficient feeding strain containing the isopropyl β-D-1-thiogalactopyranoside (IPTG)-inducible T7 polymerase, transformed with the L4440 double-T7-promoter feeding vector [10]. After inducing double-stranded RNA (dsRNA) expression from the L4440 plasmid, *E. coli* HT115 cells are fed to the ciliate to elicit RNAi till the appearance of phenotypes [6]. Using this method, RNAi against the kinase regulator Mob1 has been successfully induced in *S. coeruleus* [11]. However, the RNAi method based on *E. coli* HT115 must be induced to express dsRNA in the presence of the IPTG, and this method may not work in some ciliates (e.g., *Vorticella* [12]). Hence, a new RNAi method involving constitutive dsRNA expression without any induction is needed.

For the above reasons, we turned to the cyanobacterium *Synechocystis* sp. PCC6803 (hereafter, *Synechocystis* 6803). *Synechocystis* 6803 is a unicellular freshwater cyanobacterium that was initially isolated from a freshwater lake in 1968 [13], and has since become one of the most extensively studied cyanobacterial species. *Synechocystis* 6803 can be easily cultivated in either solid or liquid culture systems and in absence or presence of light. Its genome was fully sequenced in 1996 [14] and it can readily take up exogenous DNA via natural transformation, electroporation, ultrasonic transformation and conjugation [15]. Therefore, *Synechocystis* 6803 is considered a model organism that can be used for genetic engineering in cyanobacteria. Owing to its slow doubling time, it is more efficient to perform DNA cloning in a fast-growing host, such as *E. coli* strain DH10B [16,17], which carries the pRL443 conjugal plasmid and the pRL623 helper plasmid [18]. Generally, a shuttle vector with a broad host range and an effective gene promoter are required to create a stably replicating plasmid that will function in both *E. coli* and *Synechocystis* 6803. The RSF1010 vector [19] and the derivative, pSB2A [15], can successfully replicate in *Synechocystis* 6803. The constitutive *C-phycocyanin β* promoter (P*_cpcB_*), a slightly modified variant of the native P*_cpc_* from *Synechocystis* 6803, has been successfully applied in different *Synechocystis* strains [20,21,22]. Particularly high production of heterologous proteins in cyanobacteria have been achieved using P*_cpc560_*, a truncated version of P*_cpcB_* [22]. Hence, the strong constitutive P*_cpcB_* can control heterologous gene expression in *Synechocystis* 6803. Finally, *Synechocystis* 6803 can acquire exogenous DNA from *E. coli* via bacterial conjugation [23].

We established a novel RNAi method for the constitutive expression of exogenous gene dsRNA in *Synechocystis* 6803 and successfully applied this method to knock down a previous identified *S. coeruleus*-specific gene (named *SCSG1*), known to be associated with OA regeneration [5], and verified its function in ciliogenesis of OA regeneration.

## 2. Materials and Methods

### 2.1. Cell Culture and Growth Conditions

*S. coeruleus* (strain WHEL) [5] cells were grown at 20 °C in the dark in Modified *Stentor* Medium (MSM) [11], supplemented with *Chlorogonium elongatum* as living prey. Wild-type (WT) and the transformed strains of *Synechocystis* 6803 were grown at 30 °C in liquid or solid Blue-Green-11 (BG-11) medium at a light intensity of 40 μmoL photons m^−2^ s^−1^ in ambient air. Kanamycin (Km) was added to BG-11 medium at a concentration of 25 μg/mL or 50 μg/mL when required. Cell growth was monitored by measuring the optical density at 580 nm (OD_580_) of the cultures on a UNIC 7200 spectrophotometer (UNIC, Shanghai, China). The *E. coli* DH10B strain carrying the pRL443 conjugal plasmid and the pRL623 helper plasmid was the host for all plasmids constructed in this study. *E. coli* cells were grown in Luria-Bertani (LB) medium at 37 °C with continuous shaking. Carbenicillin (50 μg/mL), chloramphenicol (25 μg/mL) or Km (50 μg/mL) was added to the LB medium when required for the propagation of plasmids in *E. coli*.

### 2.2. RNAi Expression Vector Construction

The pSCTGA vector, containing a Km resistance cassette with mobilization (*Mob*) genes, was constructed based on the plasmid RSF1010 [24], and can successfully replicate in *Synechocystis* 6803. The pSCTGA vector was modified for use as an RNAi expression vector in *Synechocystis* 6803. Firstly, the pSCTGA vector backbone (pSCTGA-backbone) containing the replication elements, *Mob* gene, and Km resistance gene was cloning using the primer pair, pSCTGA-backbone-F and pSCTGA-backbone-R (Table S1). The forward and reverse sequences of P*_cpcB_* (FP*_cpcB_* and RP*_cpcB_*) were PCR amplified from the WT *Synechocystis* 6803 genome using the primer pairs, FP*_cpcB_*-F + FP*_cpcB_*-R and RP*_cpcB_*-F + RP*_cpcB_*-R, respectively (Table S1). The multiple cloning site (MCS) was PCR amplified from L4440 vector [10] using the primer pair, MCS-F and MCS-R (Table S1). Next, the FP*_cpcB_*, MCS and RP*_cpcB_* DNA fragments were jointed together to form a recombinant DNA fragment (FP*_cpcB_*-MCS-RP*_cpcB_*) using fusion PCR with the primer pairs, FP*_cpcB_*-F + MCS-R and FP*_cpcB_*-F + RP*_cpcB_*-R (Table S1). Finally, the FP*_cpcB_*-MCS-RP*_cpcB_* DNA fragment was joined to the pSCTGA-backbone in a correct orientation via the one-step cloning method to form the pSCT3C RNAi expression vector.

### 2.3. Plasmid Construction

To clone *Stentor* gene sequences, homologs were identified by best-reciprocal BLAST between the *S. coeruleus* WHEL [5] transcriptome and the *S. coeruleus* WM001 genome [25]. Target gene sequences were obtained by PCR amplification from *S. coeruleus* WHEL genomic DNA. PCR fragments were digested with BglII and KpnΙ and then ligated into the pSCT3C vector, which had been linearized with the same restriction enzymes. The resulting plasmids were transfected into *E. coli* DH10B cells. Additional information about the RNAi constructs used in this study according to the method of Slabodnick et al. [11] is given in Table S2.

### 2.4. Synechocystis 6803 Transformation and Identification

Transformation was performed by the conjugal transfer method [23]. Briefly, *Synechocystis* 6803 cells were centrifuged from 14 mL of a logarithmic phase (OD_580_ = ~1) culture at 13,000 rpm for 2 min. The pellet was resuspended in 1.4 mL fresh BG-11 medium. Aliquots (2 mL) of *E. coli* cells carrying various plasmids (including the empty pSCT3C plasmid) from logarithmic phase (OD_600_ = ~1) cultures were centrifuged under the same conditions. Cell pellets were resuspended in 200 μL fresh BG-11 medium, mixed with 200 μL *Synechocystis* 6803 cells, and incubated at 30 °C for 6 h at a light intensity of 40 μmoL photons m^−2^ s^−1^. Cultures were then spread onto BG-11 agar plates without antibiotics. After incubation for 24 h at 30 °C under a light intensity of 40 μmoL photons m^−2^ s^−1^, 25 μg/mL Km was added and plates were incubated at 30 °C for approximately 2 weeks at a light intensity of 40 μmoL photons m^−2^ s^−1^ until colonies appeared. Single colonies were propagated on individual BG-11 agar plates containing 25 μg/mL Km and inoculated into liquid BG-11 medium containing 50 μg/mL Km for analysis. To determine the identity of cargo plasmids in transformed *Synechocystis* 6803, transformants were subjected to bacterial colony PCR and PCR products were analyzed by 1% agarose gel electrophoresis.

### 2.5. Reverse Transcription-PCR Assay

For each sample, total RNA was extracted from a 50 mL aliquot of transformed *Synechocystis* 6803 cultured at an OD_580_ of ~1 using the TransZol Up Plus RNA kit (TransGen, Beijing, China), following the manufacturer’s instructions. Contaminating was removed with RNase-free DNase (Qiagen, Dusseldorf, Germany). Reverse transcription was carried out using M-MLV Reverse Transcriptase Assay (Invitrogen, Carlsbad, CA, USA). The resulting cDNA molecules were PCR amplified using the primer pairs shown in Table S2. All reactions were performed on the Eppendorf PCR Mastercycler (Eppendorf, Hamburg, Germany). Each reaction was performed in a total volume of 50 μL, including 8 μL cDNA, 2 μL 10 μmol/L of each primer, and 38 μL 1× BioReady ePfu Mix (BioFlux, Beijing, China). Amplification conditions were 94 °C for 5 min, followed by 29 cycles of 94 °C for 30 s, 60 °C for 30 s, and 72 °C for 1 min, and a final extension at 72 °C for another 10 min. Reverse transcription-PCR (RT-PCR) products were analyzed by 1% agarose gel electrophoresis.

### 2.6. RNAi by Feeding with Synechocystis 6803

RNAi was performed by transforming *Synechocystis* 6803 with each plasmid to allow for dsRNA expression of the target gene. Transformed *Synechocystis* 6803 cells were grown to early logarithmic phase (OD_580_ = ~0.4) and then fed to *S. coeruleus* cells that had been previously starved for 24–48 h. Every 30 *S. coeruleus* cells were fed with enriched 5 mL *Synechocystis* 6803 cells (OD_580_ = ~0.4) carrying various plasmids every 3 days for a total of 10–14 days, respectively. Controls cells were fed with either WT *Synechocystis* 6803 or *Synechocystis* 6803 transformed with empty pSCT3C plasmid.

### 2.7. Single-Cell RNA-Sequencing and Gene Expression Analysis

Single cells were isolated and their transcriptomes were amplified using Smart-Seq2 (SMARTer Ultra Low RNA kit (Art. No. 634936, Clontech, Mountain View, CA, USA) for Illumina sequencing, with an insert size of about 350 bp. RNA libraries were constructed according to the manufacturer’s protocols. Paired-end (150 bp × 2) sequencing was performed for all the single-cell RNA libraries using an Illumina NovaSeq 6000 sequencer (Illumina, San Diego, CA, USA). Raw single-cell RNA-sequencing (RNA-seq) reads were filtered by fqtools plus (https://github.com/annoroad/fqtools_plus) to trim away the reads with adapters (>5 bp adapter nucleotide) and high N ratio (>5%). Additionally, the reads were filtered by the fastq quality filter FASTX-Toolkit (-q 20, -p 80) (http://hannonlab.cshl.edu/fastx_toolkit/). Firstly, we de novo assembled a reference transcriptome by merging all RNA-seq datasets in this study using Trinity [26] with default parameters. The assembled transcript fragments served as reference transcriptome sequences. Filtered reads were mapped back to the reference transcriptome and the read count for each gene was obtained using the RSEM output integrated into Trinity [26] with default parameters. We chose the number of fragments per kilo base of transcript sequence per million base pairs (FPKM) sequenced to represent the gene expression abundance. For subsequent analysis, fold change was used in analysis of measuring change in expression level of a gene.

### 2.8. Quantitative Real-Time PCR Assay

Total RNA was extracted from 200 *S. coeruleus* cells per sample using the RNA kit (Omega, Irving, TX, USA), following the manufacturer’s instructions. After purification, RNA was treated with RNase-free DNase (Qiagen, Düsseldorf, Germany), re-purified, primed with oligo-dT, and reverse transcribed using the M-MLV Reverse Transcriptase Assay (Invitrogen, Carlsbad, CA, USA). Gene-specific primer pairs were designed for 18S SSU rRNA and *SCSG1* using Primer Premier 5.0 software (Table S3), and all reactions were performed on a CFX96 Real-Time System (Bio-Rad, Richmond, CA, USA) with three technical replicates. Each reaction was performed in a total volume of 20 μL, including 2 μL cDNA, 1 μM each primer, and 10 μL 2× AceQ qPCR SYBR Green Master Mix (Vazyme, Nanjing, China). Amplification conditions were 95 °C for 5 min, followed by 40 cycles of 95 °C for 10 s and 60 °C for 30 s, and then melt curve readings were obtained from 65 °C to 95 °C, in increments of 0.5 °C/0.05 s. All data were compared using the homogenization method.

### 2.9. Induction of OA Regeneration

*S. coeruleus* cells in the experimental and control groups were collected by mouth pipette using a Zeiss anatomical lens (Carl Zeiss AG, Oberkochen, Germany). To induce OA regeneration, cells were incubated for 1 min in a 4% urea solution [3]. This treatment caused the MBs to be sloughed from the cells, followed by replacement of the entire complement of feeding organelles. After urea treatment, cells were washed twice in cold MSM and resuspended in fresh MSM. Somatic cells (without OA) with good morphology were picked and grown under normal culture conditions for OA regeneration [5].

### 2.10. Cell Shape Imaging and Analysis

Cell shape were preliminarily observed using a Stemi 2000C anatomical lens (Carl Zeiss AG, Oberkochen, Germany). Brightfield images were collected on a BX51 microscope equipped with 4× 0.13 NA, 10× 0.3 NA, 20× 0.5 NA, 40× 0.75 NA, and 100× oil 1.3 NA objectives lenses (Olympus, Osaka, Japan). Brightfield Images were captured using an DP73 digital microscope camera (Olympus, Osaka, Japan) and analyzed using cellSens imaging software version 1.16. Fluorescence images were collected on an Axio Imager A2 microscope equipped with 5× 0.16 NA, 10× 0.3 NA, 20× 0.5 NA, 40× 0.95 NA, 63× oil 1.25 NA and 100× oil 1.3 NA objectives lenses (Carl Zeiss AG, Oberkochen, Germany). Fluorescence images were captured using a pco.edge 4.2LT sCMOS camera (PCO AG, Kelheim, Germany) and analyzed using Zen 2.3 (blue edition).

Cell shape in control and RNAi cells were compared as follows: for each cell, the widest part of the cell outline was assumed to represent the OA, and the part farthest away from the OA was assumed to represent the holdfast [11]. The size of the cell was defined as the distance between the OA and the holdfast and calculated using the scale bars of the imaging software. Live, fully extended cells were used as controls for cell shape.

## 3. Results and Discussion

### 3.1. Structure of the pSCT3C RNAi Expression Vector

**Figure 1:**
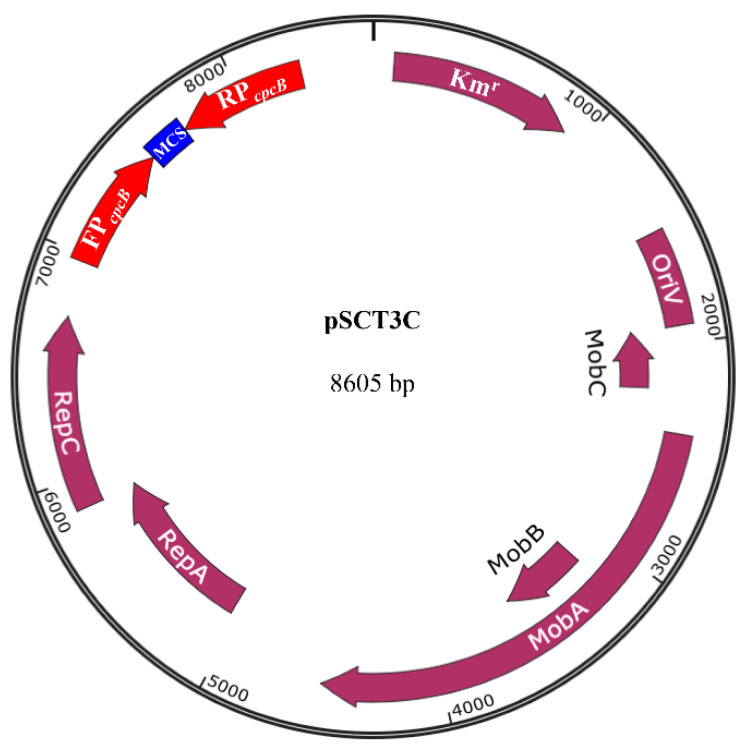
pSCT3C vector map. A DNA fragment of the target gene was amplified and cloned into the pSCT3C double-P*_cpcB_* vector, which has two *cpcB* promoters in opposite orientations flanking the multiple cloning site. The pSCT3C vector consists of the mobilization genes required for conjugative transfer and the origin for vegetative replication. The pSCT3C vector also has a selectable marker that confers Km resistance. Cloned plasmids are transformed into DH10B, an *E. coli* strain which can transfer exogenous DNA into *Synechocystis* PCC6803 via bacterial conjugation.

The 8605 bp pSCT3C plasmid (Figure 1) was assembled via a multiple-step cloning procedure and contains three essential elements: (1) the pSCTGA-backbone (7300 bp), carrying the origin for vegetative replication, the *Mob* genes [24], and a Km resistance gene for selection; (2) two *cpcB* promoters for expressing dsRNA of the exogenous gene; and (3) a MCS for inserting the DNA coding sequence of the target gene. We used the *E. coli* DH10B strain to replicate and transfer a pSCT3C plasmid containing the target gene sequence. Successful replication of the pSCT3C plasmid in *E. coli* DH10B was confirmed by PCR and sequencing.

### 3.2. Synechocystis 6803 Transformation and Gene Expression Analysis

**Figure 2:**
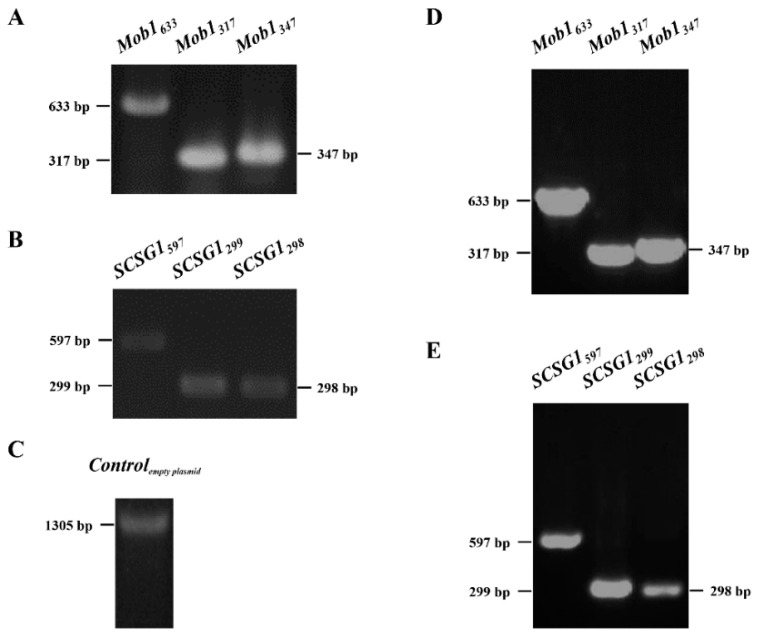
Verification of plasmid transformation into *Synechocystis* PCC6803 by bacterial colony PCR and of exogenous *SCSG1* and *Mob1* gene expression in *Synechocystis* PCC6803 transformants using RT-PCR. No DNA fragments were amplified in all controls (data not shown). (**A**) Bacterial colony PCR of *Mob1* transformants generated DNA fragments of the expected sizes (633 bp, 317 bp, and 347 bp, respectively). (**B**) Bacterial colony PCR of *SCSG1* transformants generated DNA fragments of the expected sizes (597 bp, 299 bp, and 298 bp, respectively). (**C**) Bacterial colony PCR of a negative control transformant carrying the empty pSCT3C plasmid showed the 1305 bp DNA fragment. (**D**) RT-PCR of *Mob1* transformants showed DNA fragments of the expected sizes (633 bp, 317 bp, and 347 bp, respectively). (**E**) RT-PCR of *SCSG1* transformants showed DNA fragments of the expected sizes (597 bp, 299 bp, and 298 bp, respectively).

For both *Mob1* and *SCSG1*, three expression plasmids were constructed (to express three different regions of each gene): Mob1_633_, Mob1_317_, and Mob1_347_; and SCSG1_597_, SCSG1_299_, and SCSG1_298_ plasmid (Table 1). The empty pSCT3C plasmid without any exogenous gene was used as the control. Each plasmid was introduced into *Synechocystis* 6803 and successful transformation was verified by bacterial colony PCR using the primer pairs listed in Table S2. The WT strain was used as the control. PCR products of the expected sizes were obtained from the Mob1_633_, Mob_317_, Mob1_347_, SCSG1_597_, SCSG1_299_, and SCSG1_298_ transformants (Figure 2A,B). As expected, a transformant carrying the empty pSCT3C plasmid (primer pair FP*_cpcB_*-F + RP*_cpcB_*-R) generated PCR products of 1305 bp (Figure 2C). For WT untransformed control cells, no PCR products were obtained. We successfully transformed *Synechocystis* 6803 cells with *Mob1* and *SCSG1* gene fragments (shown in Figure 2A–C). This result also suggests that the pSCT3C RNAi expression vector can replicate in *Synechocystis* 6803.

To determine the expression of *Mob1* and *SCSG1* in the transformants, we analyzed the transcription of *Mob1* and *SCSG1* gene by RT-PCR. DNA fragments of the expected sizes were amplified in *Mob1* and *SCSG1* transformants and not in the WT control (Figure 2D,E). This result confirms that the *Mob1* and *SCSG1* gene can be expressed in *Synechocystis* 6803.

### 3.3. Successful Knockdown of Mob1 Supports the Applicability of RNAi by Synechocystis 6803 Feeding

**Figure 3:**
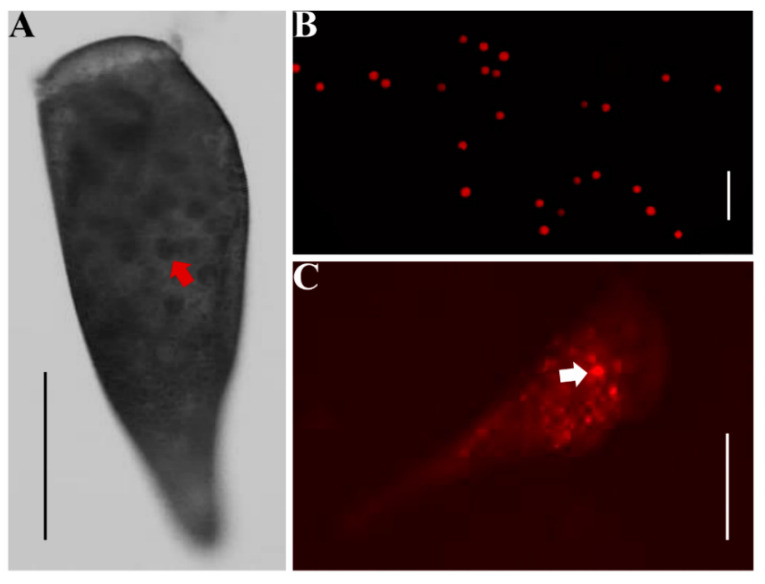
Ingestion of *Synechocystis* PCC6803 by *S. coeruleus*. (**A**) Brightfield microscopy image of a live *S. coeruleus* cell with *Synechocystis* PCC6803 stored in its food vacuoles. The red arrow indicates a food vacuole. Scale bar: 100 μm. (**B**) Autofluorescence microscopy image of live *Synechocystis* PCC6803 cells. Scale bar: 50 μm. (**C**) Autofluorescence microscopy image of a live *S. coeruleus* cell with *Synechocystis* PCC6803 stored in its food vacuoles. The white arrow indicates a food vacuole. Scale bar: 100 μm.

To determine whether transformed *Synechocystis* 6803 can mediate RNAi by feeding in *S. coeruleus*, we first checked whether *Synechocystis* 6803 cells are ingested by *S. coeruleus*. We found that many food vacuoles in *S. coeruleus* cells contained the *Synechocystis* 6803 cells (Figure 3), suggesting that *Synechocystis* 6803 could be a good food resource and can be used as RNAi system at least in *Stentor*.

**Figure 4:**
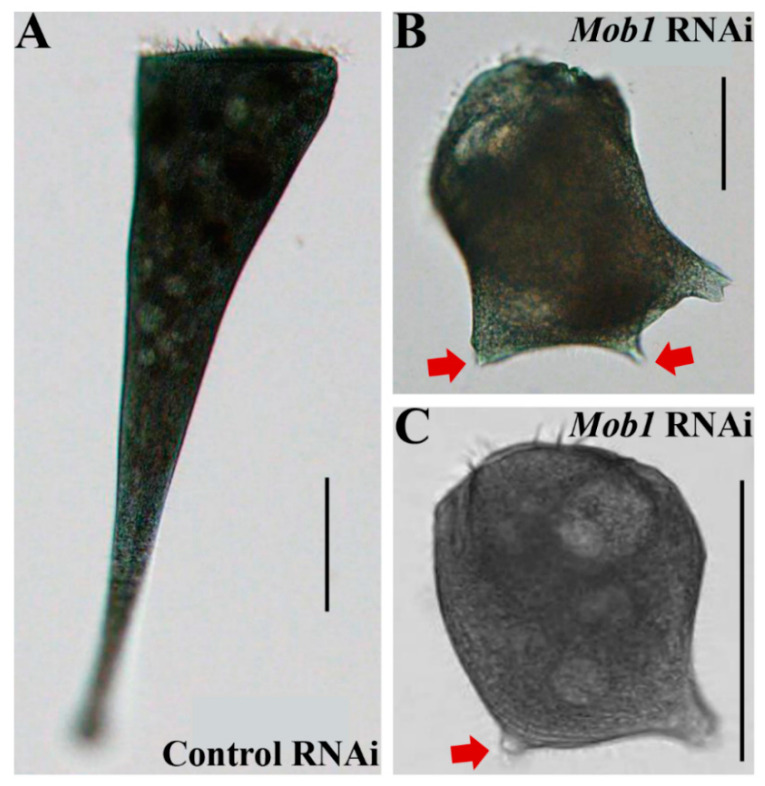
*Mob1* RNAi resulted in aberrant cell polarity in *S. coeruleus*. (**A**,**B**) Representative brightfield microscopy images of cells treated with control RNAi (**A**) and *Mob1* RNAi (**B**) by *Synechocystis* 6803 feeding for 10 days. *Mob1* RNAi resulted in the development of two ectopic posterior poles (red arrows) in cell under normal growth conditions. Scale bars: 100 μm (**A**) and 50 μm (**B**). (**C**) Representative brightfield microscopy image of a cell treated with *Mob1* RNAi by *Synechocystis* 6803 feeding for 14 days. *Mob1* RNAi resulted in the development of an ectopic posterior pole (red arrow) during OA regeneration. Scale bars: 100 μm.

To further test the applicability of our method, we next checked whether RNAi of *Mob1* by *Synechocystis* 6803 works in *S. coeruleus*. *Mob1* encodes a patterning protein required for *Stentor* morphogenesis. In a previous study, the *Mob1* was knocked down using the *E. coli* HT115 method and the phenotypes were determined [11]. Therefore, we performed *Mob1* RNAi in *S. coeruleus* by *Synechocystis* 6803 feeding. As Mob1 plays a key role in defining polarity and regulating polarized cell growth during both normal development and regeneration [11], we expected to observe phenotypes related to cell polarity. Compared with WT *S. coeruleus* cells under normal growth conditions (Figure 4A), *Mob1* RNAi led to an aberrant cell shape after 10 days of feeding with *Synechocystis* 6803 including the Mob1_317_ RNAi vector (Table 1) that included defective OA morphogenesis, loss of the characteristic “trumpet” shape, and two ectopic posterior poles (Figure 4B). After 14 days of RNAi feeding with *Synechocystis* 6803 including the Mob1_633_ RNAi vector (Table 1), the similar phenotypes were observed in OA regeneration of *Stentor*, except that only one ectopic posterior pole could be seen (Figure 4C). As a result, the *Mob1* RNAi efficiency is approximately 5.9% in the *Mob1*_317_ and *Mob1*_633_ RNAi cells, while we did not observe the *Mob1* RNAi phenotype shown in the *Mob1*_347_ RNAi cells (Table 1). In previously reported in another *S. coeruleus* strain, 10% of cells failed to reestablish normal cell proportions after regenerating OA/holdfast in *Mob1* RNAi cells fed with *E. coli* HT115 [11]. Although our RNAi efficiency are less than the previous one, the *Mob1* RNAi phenotypes are similar to those of the previous reports [11]. It is suggested that our RNAi method is reliable.

### 3.4. Gene Expression Profiles and Function Analysis of SCSG1 during OA Regeneration

**Figure 5:**
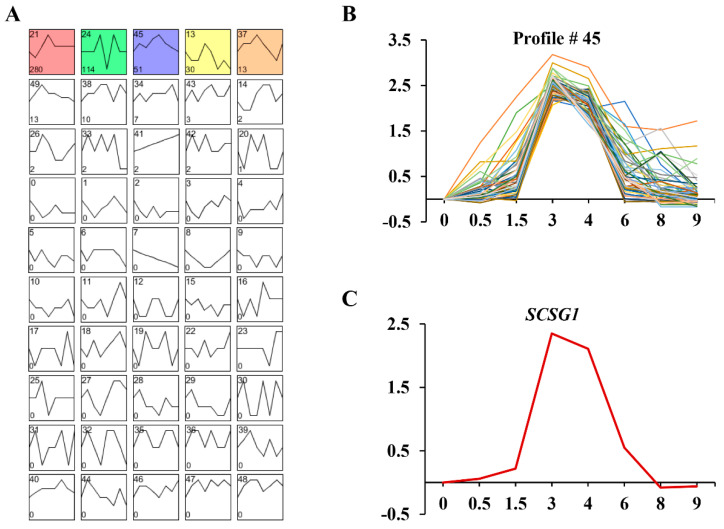
(**A**) The clustering analysis of 532 upregulated DEGs with maximum expression at the stage of the first OA cilia appearance during OA regeneration in *S. coeruleus*. Five expression patterns (No. 21, 24, 45, 37, and 13) of genes showed statistically significant difference (*p* < 0.00001) (colored boxes). (**B**) The time-series analysis of 51 upregulated DEGs in No. 45 pattern. The x-axis shows the time points, and the y-axis shows the time series of gene expression levels. (**C**) Gene expression profiles of *SCSG1 gene* during OA regeneration in *S. coeruleus*. The x-axis shows the time points, and the y-axis shows the time series of gene expression levels.

In previous single-cell RNA-seq study, we obtained 3223 upregulated differentially expressed genes (DEGs) during OA regeneration in *S. coeruleus* WHEL [5]. Among them, expression of 532 DEGs peaked at 3 h, the time of OA cilia appearance. The 532 DEGs were clustered into 50 profiles using STEM [27] (Figure 5A), and genes were significantly enriched in five profiles (No. 21, 24, 45, 37, and 13) (Figure 5A). The profile No. 45 (51 genes) shows high expression level at 3–4 h, the very important stage of ciliogenesis in OA regeneration (Figure 5B). Therefore, we focused on these 51 genes, and found these genes including centrins, kinesins, and protein kinases which may involve in cilia growth, indicating the important functions of these genes during ciliogenesis. In these genes, we identified a highly expressed gene (SCOERU2802901, Figure 5C) which do not have any homolog in other ciliates, and thus named as *S. coeruleus*-specific gene (*SCSG1*).

### 3.5. SCSG1 RNAi Illustrates Its Essential Function in OA Regeneration

**Figure 6:**
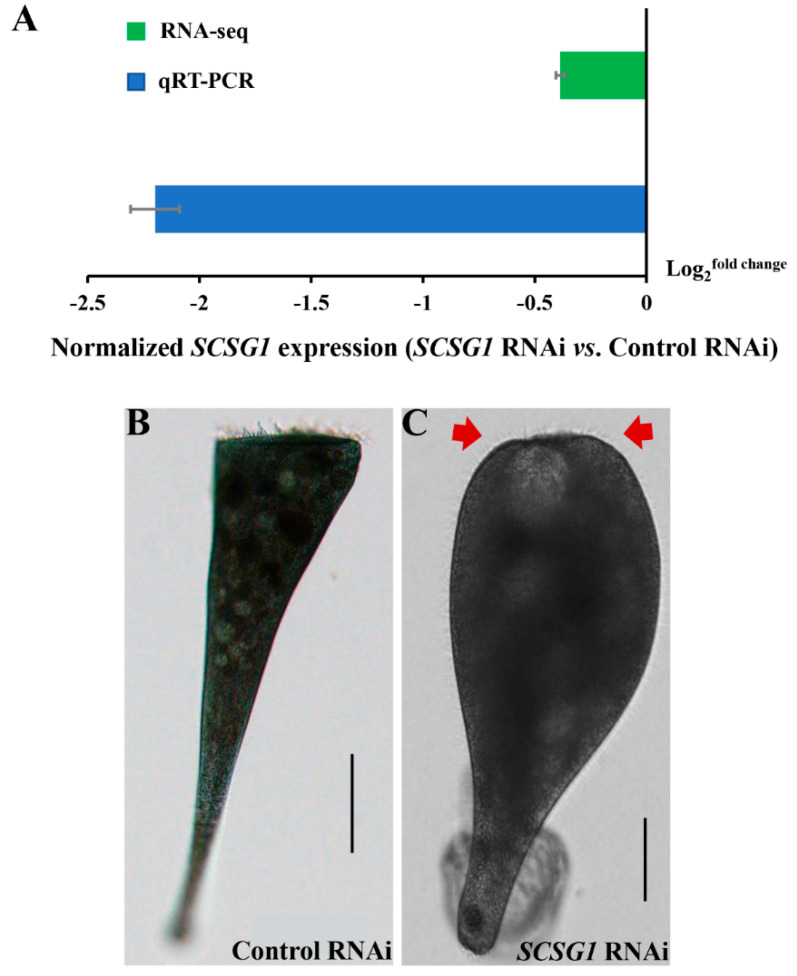
*SCSG1* RNAi resulted in aberrant cell morphology during OA regeneration in *S. coeruleus*. (**A**) RNA-seq and qRT-PCR data showing down-regulated expression of *SCSG1* normalized to *18S* expression in control and *SCSG1* RNAi cells, respectively. (**B**,**C**) Brightfield microscopy images of *S. coeruleus* cells treated with control and *SCSG1* RNAi, showing normal (**B**) and aberrant (**C**) cell morphology without regenerated OA (red arrows). Scale bars: 100 μm (**B**) and 50 μm (**C**).

Considering the high expression level of *SCSG1* in cilia growth, we expect a phenotype that would affect the new OA formation. Therefore, we created RNAi vectors targeting various *SCSG1* sequences to determine gene function. After feeding with *Synechocystis* 6803 for 14 days, we measured *SCSG1* expression using both single-cell RNA-seq and quantitative real-time PCR (qRT-PCR). For single-cell RNA-seq, we subjected approximately 300 cells with normal morphology to urea treatment and then observed the morphology of cells every 1 h until OA regeneration was complete. Before urea treatment, a single cell was randomly collected to serve as a negative control for RNA-seq for baseline *SCSG1* expression. For qRT-PCR analysis, we collected approximately 300 cells. The RNA-seq and qRT-PCR results showed that *SCSG1* expression in *S. coeruleus* was significantly knocked down following *Synechocystis* 6803 feeding (Figure 6A). After OA regeneration was induced by urea treatment, about 5.2% of *SCSG1* RNAi cells fed with *Synechocystis* 6803 including the SCSG1_299_ RNAi vector did not regenerate a new OA (Table 1, Figure 6B,C). The time course of morphological changes during OA regeneration in *SCSG1* RNAi cells shown in Figure S1. These cells did not form OA cilia and adopted an aberrant shape consistent with wound healing. After a few days, cells that failed to regenerate an OA died because they could not ingest food. However, we did not observe phenotype in the *SCSG1*_597_ and *SCSG1*_298_ RNAi cells (Table 1). These results demonstrated that *SCSG1* is required for ciliogenesis during OA regeneration in *S. coeruleus*. Cilia/flagella-associated protein 20 (CFAP20) was identified in the OA proteome of *S. coeruleus* [5]. It is a cilium/flagellum-specific protein involved in axonemal structure organization and motility in *Paramecium* [28] and *Chlamydomonas* [29], and regulates cilia size and morphology in *Drosophila* [30]. Notably, the expression pattern of *CFAP20* is similar to the *SCSG1* transcription pattern during OA regeneration in *S. coeruleus* we previously determined by single-cell transcriptome analysis [5], with both peaks of expression at the first appearance of OA cilia. However, SCSG1 was not found in the OA proteome of *S. coeruleus*. Moreover, *SCSG1* RNAi did not result in defective morphogenesis during normal growth. This further suggests that *SCSG1* may be a cilium-specific gene that is induced by OA regeneration and regulates cilia growth during OA regeneration in *S. coeruleus*.

In summary, we developed a novel RNAi method in *Stentor* that may be applicable to other ciliates that can use cyanobacteria as food. Using the new RNAi method, we confirmed that *SCSG1* has an essential function in ciliogenesis during OA regeneration in *S. coeruleus*.

## Figures and Tables

**Table 1:** Efficiency of RNAi constructs targeted different regions of each gene in this study.

Gene	Construct Name	Length (bp)	Target Region in CDS	Sample Size (Cell Number)	RNAi Efficiency
*Mob1* CDS length: 675 bp	Mob1_633_	633	40–672 bp	51	5.9%
	Mob1_317_	317	40–356 bp	34	5.9%
	Mob1_347_	347	326–672 bp	40	0
*SCSG1* CDS length: 597 bp	SCSG1_597_	597	1–597 bp	43	0
	SCSG1_299_	299	1–299 bp	58	5.2%
	SCSG1_298_	298	300–597 bp	57	0

## Data Availability

The sequencing data is freely available at http://bigd.big.ac.cn/gsa/s/J4yTU1HH.

## Supplementary Materials

The following are available online at https://www.mdpi.com/2076-2607/9/1/176/s1. Figure S1: Selected images from a time course of morphological changes in control and *SCSG1* RNAi cells during OA regeneration in *S. coeruleus*. Control and *SCSG1* RNAi cells were observed after the start of OA regeneration and imaged every 2 h. (A,B) Brightfield microscopy images of *S. coeruleus* cells treated with control and *SCSG1* RNAi, showing normal cell morphology of OA regeneration (A) (black arrows) and aberrant cell morphology without regenerated OA (B) (red arrows). Scale bars: 100 μm (A) and 50 μm (B). Table S1: Primers with specific adaptor sequences information for the PCR amplify with homologous recombination method; Table S2: Information of RNAi constructs in this study. Their lengths and relative positions within the coding sequence, and primers with restriction enzyme cutting site and protective base used to amplify the sequences from genomic samples; Table S3: Primers used for qRT-PCR analysis.
