# Supplementary material for: RNA Interference by Cyanobacterial Feeding Demonstrates the SCSG1 Gene Is Essential for Ciliogenesis during Oral Apparatus Regeneration in Stentor
Source: Microorganisms. 2021 Jan 15;9(1):176. doi: 10.3390/microorganisms9010176 (PMC7830263; doi:10.3390/microorganisms9010176)
Supplement: Supplementary file 1 [file microorganisms-09-00176-s001.zip › microorganisms-1059190-supplementary/microorganisms-1059190-supplementary-2nd resubmit/Supplementary Table 1.docx]

**Supplementary Table S1.** Primers with specific adaptor sequences information for the PCR amplify with homologous recombination method.

| **Primer name** | **Sequence information** |
| --- | --- |
| pSCTGA-backbone-F | AGCACTAGCGTCGGT agcgcttggagccatcccca |
| pSCTGA-backbone-R | CCAAAAAAAAACCCC gccgaagcggggagatcctttt |
| FP*_cpcB_*-F | GGGGTTTTTTTTTGG acctgtagagaagagtccctgaatatcaaaatgg |
| FP*_cpcB_*-R | TGAATTAATCTCCTA cttgactttatgagttgggattttcttaaacacaatt |
| RP*_cpcB_*-F | TGAATTAATCTCCTA cttgactttatgagttgggattttcttaaacacaa |
| RP*_cpcB_*-R | ACCGACGCTAGTGCT acctgtagagaagagtccctgaatatcaaaatgg |
| MCS-F | TAGGAGATTAATTCA gagaccggcagatctgatatcatcgatg |
| MCS-R | TAGGAGATTAATTCA gcgaattgggtaccgggccc |

Uppercase letters indicate the homologous sequences and lowercase letters indicate the primer sequences.
