# Supplementary material for: RNA Interference by Cyanobacterial Feeding Demonstrates the SCSG1 Gene Is Essential for Ciliogenesis during Oral Apparatus Regeneration in Stentor
Source: Microorganisms. 2021 Jan 15;9(1):176. doi: 10.3390/microorganisms9010176 (PMC7830263; doi:10.3390/microorganisms9010176)
Supplement: Supplementary file 1 [file microorganisms-09-00176-s001.zip › microorganisms-1059190-supplementary/microorganisms-1059190-supplementary-2nd resubmit/Supplementary Table 3.docx]

**Supplementary Table S3.** Primers used for qRT-PCR analysis.

| **Primer name** | **Sequence information** |
| --- | --- |
| 18S-F | aagaacggccatgcaccacc |
| 18S-R | ttcagcatcttccgagaaatcaaagt |
| SCSG1-F | tagcggcaagcggtagtgca |
| SCSG1-R | tggggctggcgctggtt |
