# Supplementary figures and images for: RNA Interference by Cyanobacterial Feeding Demonstrates the SCSG1 Gene Is Essential for Ciliogenesis during Oral Apparatus Regeneration in Stentor
Source: Microorganisms. 2021 Jan 15;9(1):176. doi: 10.3390/microorganisms9010176 (PMC7830263; doi:10.3390/microorganisms9010176)

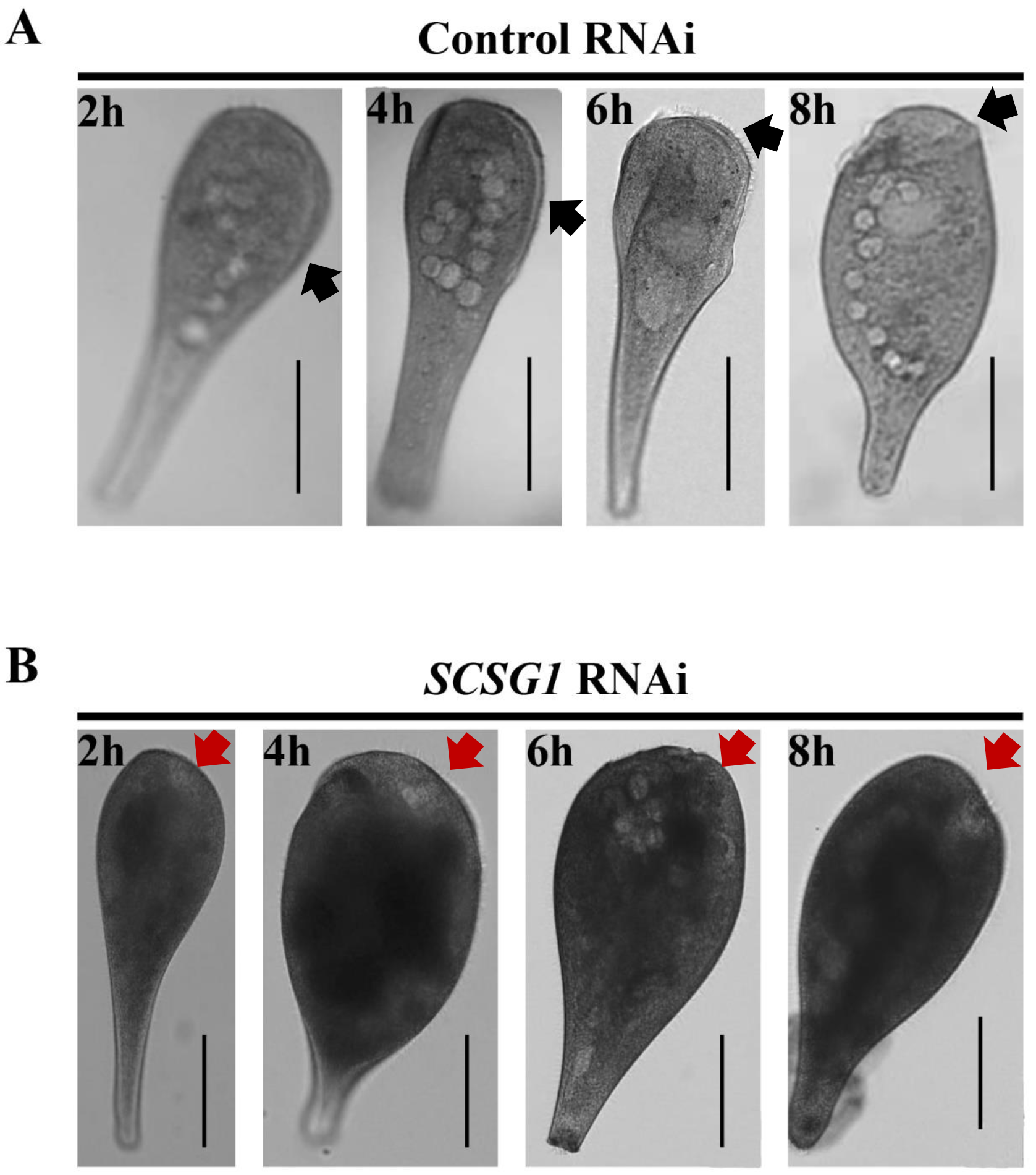

Supplement: Supplementary file 1 [file microorganisms-09-00176-s001.zip › microorganisms-1059190-supplementary/microorganisms-1059190-supplementary-2nd resubmit/Supplementary Figure 1.tif]
